# Visceral leishmaniasis in the state of Sao Paulo, Brazil: spatial and space-time analysis

**DOI:** 10.1590/S1518-8787.2016050005965

**Published:** 2016-08-08

**Authors:** Marisa Furtado Mozini Cardim, Marluci Monteiro Guirado, Margareth Regina Dibo, Francisco Chiaravalloti

**Affiliations:** I Faculdades Adamantinenses Integradas. Adamantina, SP, Brasil; II Laboratório de Vetores. Superintendência de Controle de Endemias. São José do Rio Preto, SP, Brasil; III Divisão de Programas Especiais. Superintendência de Controle de Endemias. São Paulo, SP, Brasil; IV Departamento de Epidemiologia. Faculdade de Saúde Pública. Universidade de São Paulo. São Paulo, SP, Brasil

**Keywords:** Visceral leishmaniasis, epidemiology, Spatial Analysis, Spatio-Temporal Analysis, Space-Time Clustering, Epidemiology, Descriptive

## Abstract

**OBJECTIVE:**

To perform both space and space-time evaluations of visceral leishmaniasis in humans in the state of Sao Paulo, Brazil.

**METHODS:**

The population considered in the study comprised autochthonous cases of visceral leishmaniasis and deaths resulting from it in Sao Paulo, between 1999 and 2013. The analysis considered the western region of the state as its studied area. Thematic maps were created to show visceral leishmaniasis dissemination in humans in the municipality. Spatial analysis tools Kernel and Kernel ratio were used to respectively obtain the distribution of cases and deaths and the distribution of incidence and mortality. Scan statistics were used in order to identify spatial and space-time clusters of cases and deaths.

**RESULTS:**

The visceral leishmaniasis cases in humans, during the studied period, were observed to occur in the western portion of Sao Paulo, and their territorial extension mainly followed the eastbound course of the Marechal Rondon highway. The incidences were characterized as two sequences of concentric ellipses of decreasing intensities. The first and more intense one was found to have its epicenter in the municipality of Castilho (where the Marechal Rondon highway crosses the border of the state of Mato Grosso do Sul) and the second one in Bauru. Mortality was found to have a similar behavior to incidence. The spatial and space-time clusters of cases were observed to coincide with the two areas of highest incidence. Both the space-time clusters identified, even without coinciding in time, were started three years after the human cases were detected and had the same duration, that is, six years.

**CONCLUSIONS:**

The expansion of visceral leishmaniasis in Sao Paulo has been taking place in an eastbound direction, focusing on the role of highways, especially Marechal Rondon, in this process. The space-time analysis detected the disease occurred in cycles, in different spaces and time periods. These meetings, if considered, may contribute to the adoption of actions that aim to prevent the disease from spreading throughout the whole territory of São Paulo or to at least reducing its expansion speed.

## RESUMO

**OBJETIVO:**

Avaliar, no espaço e espaço-tempo, a ocorrência da leishmaniose visceral em humanos no estado de São Paulo, Brasil.

**MÉTODOS:**

Considerou-se como população de estudo os casos autóctones e óbitos por leishmaniose visceral em humanos ocorridos em São Paulo entre 1999 e 2013 e, como área de estudo, a região oeste do estado. Construíram-se mapas temáticos que mostraram a disseminação da leishmaniose visceral em humanos nos municípios. Ferramentas de análise espacial Kernel e razão Kernel foram utilizadas para obter, respectivamente, a distribuição dos casos e óbitos e a distribuição da incidência e mortalidade. Utilizaram-se estatísticas de varredura para identificar aglomerados espaciais e espaço-temporais de casos e óbitos.

**RESULTADOS:**

Os casos de leishmaniose visceral em humanos, no período de estudo, ocorreram na parte ocidental de São Paulo e sua expansão territorial seguiu principalmente o curso da rodovia Marechal Rondon, no sentido oeste-leste. As incidências foram caracterizadas como duas sequências de elipses concêntricas com intensidade decrescente. A primeira, com maior intensidade, teve epicentro no município de Castilho (cruzamento da rodovia Marechal Rondon com a divisa com o estado de Mato Grosso do Sul) e a segunda, em Bauru. A mortalidade apresentou comportamento similar ao da incidência. Os aglomerados espaciais e espaço-temporais de casos coincidiram com as duas áreas de maiores incidências. Ambos os aglomerados espaço-temporais identificados, mesmo sem coincidirem temporalmente, tiveram início após três anos da detecção dos casos humanos e tiveram a mesma duração, seis anos.

**CONCLUSÕES:**

A expansão da leishmaniose visceral em São Paulo vem ocorrendo no sentido oeste-leste, com destaque ao papel das rodovias, especialmente a Marechal Rondon, neste processo. A análise espaço-temporal detectou, em diferentes espaços e períodos de tempo, ciclicidade na ocorrência da doença. Esses encontros, se considerados, podem colaborar para a adoção de ações que visem a evitar que a doença se espalhe por todo o território paulista ou, no mínimo, diminuir sua velocidade de expansão.

## INTRODUCTION

It is estimated that 350 million people worldwide are under risk of acquiring leishmaniasis in its various clinical forms, and two million cases are expected to occur every year^a^. Brazil is among the five countries that sum up 90.0% of leishmaniasis cases, along with Bangladesh, India, Nepal, and Sudan.

In the Americas, the most serious form of the disease is visceral leishmaniasis, which occurs in Central and South America, with most cases being notified in Brazil^b^. It is an anthropozoonosis whose agent is *Leishmania (Leishmania) infantum chagasi*, whose main vector is *Lutzomyia longipalpis*, and whose main reservoir is the household dog. It is a serious disease that leads to death if not properly treated.

Visceral leishmaniasis has been observed to quickly spread across territories and urban centers in Brazil^13^ ^,^ ^20^. In the state of Sao Paulo, the time series of visceral leishmaniasis cases was observed to start in 1999, having the western region as its gateway and spreading to other regions, a process that is still in progress^7^. Better understanding this process and identifying its determining factors may facilitate the adoption of effective surveillance and control initiatives, thus preventing visceral leishmaniasis from spreading throughout Sao Paulo’s whole territory, or, at least, reducing its expansion speed.

Such expansion has been observed to occur both in space and time, with an increase in the number of affected municipalities, cases, and deaths^7^. Using geographic information systems (GIS) and spatial analysis tools allows analyzing this process and enables us to understand the spatial patterns of the disease distribution, to identify risk areas and possible associated factors, and to indicate priority areas for conducting surveillance and control initiatives^19^ ^,^ ^21^ ^,^ ^23^. Thus, this study aimed to describe the occurrence of human visceral leishmaniasis (HVL) in space and in space-time in the state of Sao Paulo, between 1999 and 2013.

## METHODS

**Figure 1:**
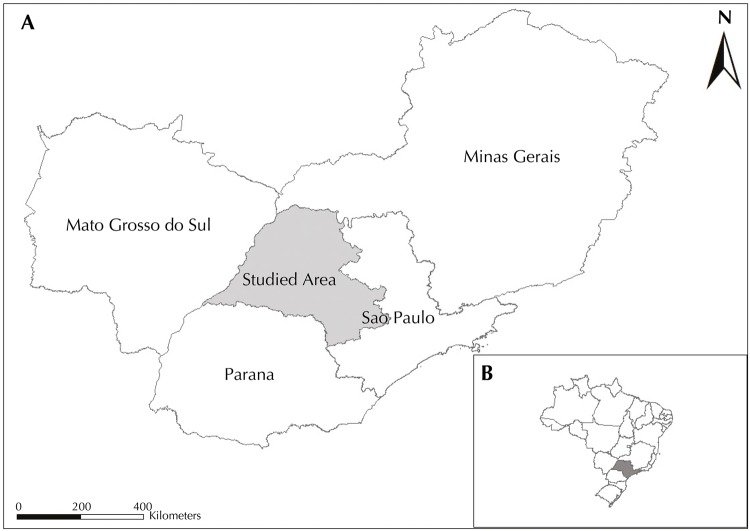
Studied area, comprising the health care regions of Aracatuba, Bauru, Marilia, Presidente Prudente, and Sao Jose do Rio Preto, located in the state of Sao Paulo, and Sao Paulo’s neighboring states (Minas Gerais, Mato Grosso do Sul, and Parana) (A) and the location of Sao Paulo in Brazil (B).

In this descriptive and ecological study, we evaluated the autochthonous cases and HVL-related deaths reported in the state of Sao Paulo between 1999 and 2013 in the health care regions of Aracatuba, Bauru, Marilia, Presidente Prudente, and Sao Jose do Rio Preto, which are the places where the disease occurs. This area has 316 municipalities (49.0% of the municipalities in the state) and is located in the western portion of Sao Paulo (Figure 1). In 2010, it had 5,609,345 inhabitants and its main activity is agribusiness. Its municipalities had, in 2010, an average human development index of 0.739 (high level), which ranged from 0.639 (medium level) to 0.862 (very high level), with 91.5% of the population in the area living in urban areas.

The information on HVL cases was obtained from the *Sistema de Informação de Agravos de Notificação* (SINAN –Information System on Diseases of Compulsory Declaration). A confirmed human case was considered to be any suspected visceral leishmaniasis case whose parasites were identified in a culture or by a parasitology test. Also, these cases included the ones taking place in an area of occurrence of the disease, with clinical signs and positive response to treatment, after all remaining diagnoses had been ruled out^c^. The populations in the municipalities, their maps, or the maps of roads in the state were obtained from the Brazilian Institute of Geography and Statistics (IBGE). The maps were georreferenced by Lat/Long SAD-69 system. The highways were classified as radial (connections with the state capital) and transverse (connecting state locations without crossing the capital).

We identified the years when the first autochthonous cases took place in each municipality with HVL cases. The Kernel tool was used to produce maps of death cases due to HVL for all the studied area and period, from the allocation of total cases and deaths in the municipalities’ centroids, thus obtaining the distribution of cases and deaths. The tool was also used to produce the distribution of the number of inhabitants per area, based on information on the population of the municipalities. The distribution of cases per area, as divided by the distribution of inhabitants per area, called Kernel ratio, has produced a map with HVL incidence rates for all areas and studied periods. A similar procedure has produced a map of mortality rates due to HVL. We delimited, in the incidence and mortality maps, regions whose rates had values between the median and the maximum value, to which the related highways were assigned.

To identify the spatial and space-time clusters of cases and HVL-related deaths, case and death databases were created, with information on the municipalities’ populations and geographical coordinates of their centroids. To identify purely spatial clusters, the discrete Poisson equation was used, with the following conditions: no geographical overlaying of clusters; maximum cluster size of 50.0% of the exposed population; round-shaped clusters; significance level of 0.05; and 999 replications.

The same conditions of the spatial analysis were used to identify the space-time clusters, considering a maximum time size of 50.0% of the studied period and time precision expressed in years^14^. We obtained clusters and their respective relative risks (RR), which were controlled by the municipalities’ populations, and thematic maps showing the spatial and space-time clusters for cases and deaths.

The study was approved by the Research Ethics Committee of Sao Jose do Rio Preto Medical School (Protocol 381/2009).

## RESULTS

Between 1999 and 2013, 2,324 cases and 200 HVL-related deaths were reported in Sao Paulo. They were concentrated in five health care regions and affected 80 municipalities, which correspond, respectively, to incidence and mortality of 2.8 cases and 0.2 deaths per 100 thousand inhabitant-years and lethality of 8.6%. Among the reported cases, 97.4% took place in urban areas and 2.4% in rural areas (0.2% of them did not have these data), which respectively corresponds to incidences of 3.0 and 0.8 cases per 100 thousand inhabitant-years.

Men were found to have higher incidences than women, with values respectively equal to 3.5 and 2.2 cases per 100 thousand inhabitant-years. The age range of highest incidence was zero to four years (12.5 cases per 100 thousand inhabitant-years), followed by ranges 60 years or older, 40 to 59, 5 to 19, and 20 to 29, with values between 2.7 and 1.6 cases per 100 thousand inhabitant-years. It was not possible to evaluate the occupation data of people with HVL because this item was not always complete in the reporting sheets (61.0% without this item).

**Figure 2:**
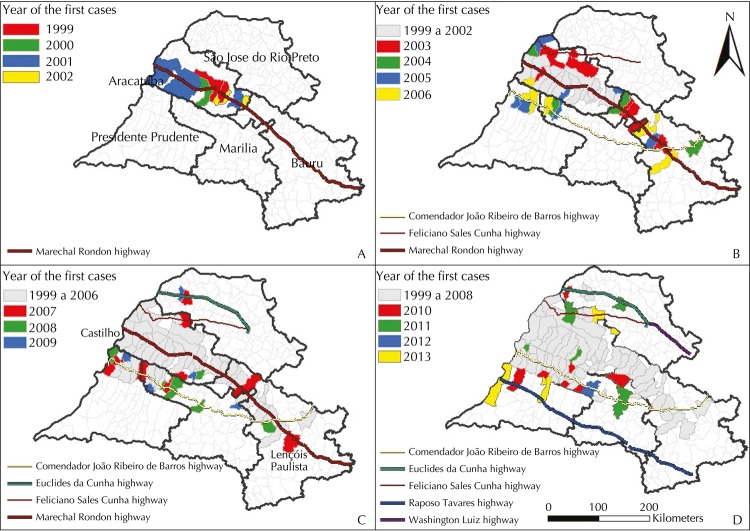
Years when the first autochthonous cases of human visceral leishmaniasis were reported in the health care regions of Aracatuba, Bauru, Marilia, Presidente Prudente, and Sao Jose do Rio Preto, state of Sao Paulo, 1999 to 2013.

Figure 2 (A) shows the first municipalities to report autochthonous cases in 1999, Aracatuba and Birigui, in the health care region of Aracatuba. By 2002, the expansion of the disease had been restricted to the municipalities in this region, of which Bilac is the only one Marechal Rondon highway does not intersect. On the other hand, among the 16 municipalities in the health care region of Aracatuba that are on the path of this highway, only four (25.0%) were not found to have autochthonous HVL occurrence between 1999 and 2002.

In the period between 2003 and 2007, the disease followed its expansion course eastwards (Figure 2, B and C). In the health care region of Bauru, the first municipalities affected were Bauru, Guaicara, and Lins, and in the region of Marilia, in 2003, Guaranta was the first one – all of these are on the way of Marechal Rondon highway. Between 2004and 2007, seven municipalities in this region were found to have HVL cases (Promissao, in 2004; Avai, in 2005; Agudos and Pirajuí, in 2006; and Cafelandia and Lencois Paulista, in 2007) – all on the path of the highway. Among the 26 municipalities on the path of Marechal Rondon that were located within the studied area, between the far west area (Castilho) and Lencois Paulista (Figure 2, C), only three were not found to have HVL cases between 1999 and 2007 (Rubiacea and Glicerio, in the health care region of Aracatuba, and Presidente Alves, the health care region of Bauru).

As of 2003, cases of the disease were detected in new municipalities north (health care region of Aracatuba) and south (regions of Marilia and Presidente Prudente) of the Marechal Rondon highway. These municipalities are adjacent to municipalities that had already been observed to have human transmission cases and which were intercepted by other radial highways parallel to Marechal Rondon (Feliciano Salles Cunha in the North and Comendador Joao Ribeiro de Barros in the South).

The disease reached the health care region or Presidente Prudente in 2005 (Figure 2, B), with cases reported in Dracena and Ouro Verde. Sao Jose do Rio Preto was the last affected region, with cases reported as of 2007 (Figure 2, C). Figure 2 (D) represents the full spread of visceral leishmaniasis in the five regions during the studied period. Between 1999 and 2013, among the 32 municipalities in the studied area that are on the path of Marechal Rondon highway, only seven (22.0%) were not found to have autochthonous HVL cases.

**Figure 3:**
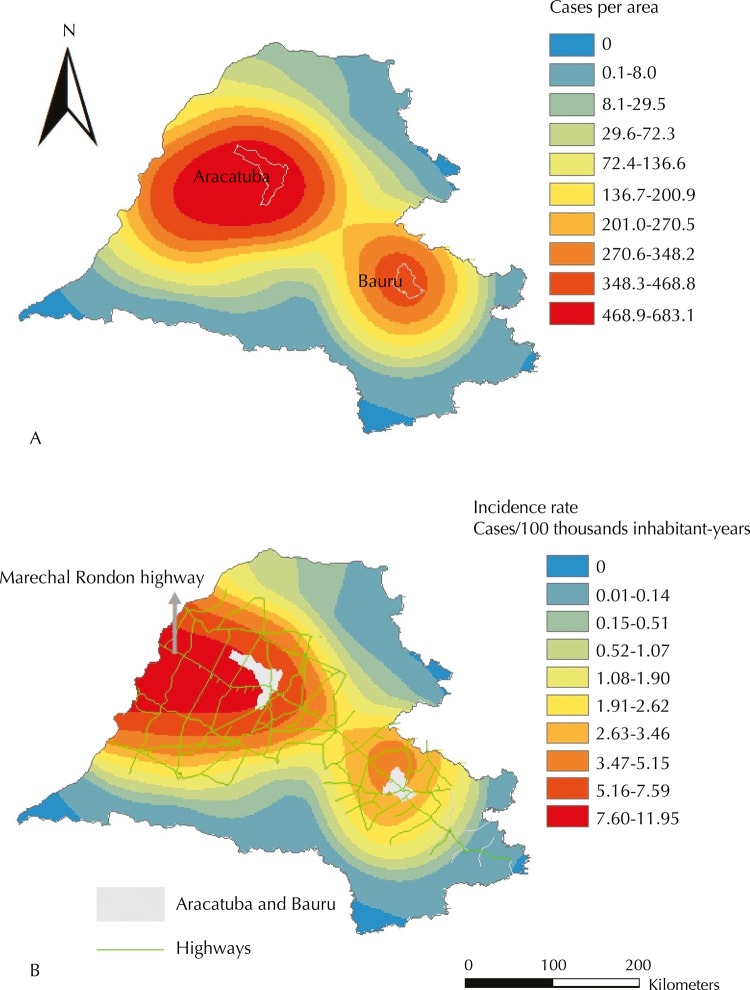
Kernel map showing the number of human visceral leishmaniasis cases per area (A); Kernel ratio map showing the incidence rate of human visceral leishmaniasis and highways, highlighting the Marechal Rondon highway (B). Health care regions of Aracatuba, Bauru, Marilia, Presidente Prudente, and Sao Jose do Rio Preto, state of Sao Paulo,1999 to 2013.

Figure 3 (A and B) respectively shows the Kernel map with the number of HVL cases per area and the Kernel ratio map, with HVL incidence, during the whole studied period. Figure 3 (B) also shows the network of highways in the area whose incidences were greater or equal to 1.9 cases per 100 thousand inhabitant-years (median).

The distribution of cases per area may be characterized by two sequences of concentric ellipses of decreasing intensities from their epicenters. The first one has higher intensity and range than the second one, and its epicenter is located at a point west of Aracatuba. The second sequence has Bauru as its epicenter.

HVL incidence, by taking into account the population sizes of the municipalities, had a distinct behavior from the distribution of cases per area, especially in the health care region of Aracatuba and surroundings, the highest rates in the whole studied area. Their distribution was characterized by a sequence of half ellipses with decreasing intensities, whose main axis may be represented by the highway that longitudinally crosses the health care region of Aracatuba (Marechal Rondon highway). The epicenter of this distribution is supposedly in the intersection between this highway and the border between the states of Mato Grosso do Sul and Sao Paulo, and it may be represented by the municipality of Castilho, which had confirmed autochthonous HVL cases in 2001.

In the health care region of Bauru, we found the incidence rates had smaller intensities than the ones of Aracatuba. Similar to the HVL cases per area, its behavior (Figure 3) was also a sequence of concentric ellipses with decreasing intensities, with their main axis being represented by Marechal Rondon highway and their epicenter being in a location next to Bauru, in the northeast direction.

**Figure 4:**
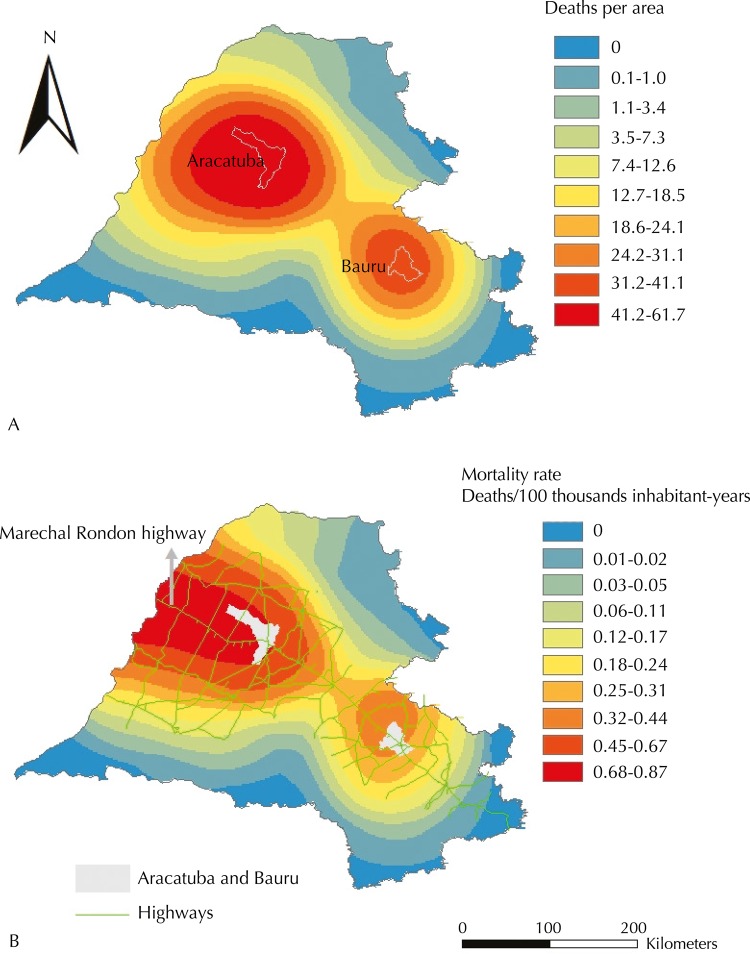
Kernel map showing the number of deaths from human visceral leishmaniasis per area (A); Kernel ratio map showing the mortality rate from human visceral leishmaniasis and highways, highlighting the Marechal Rondon highway (B). Health care regions of Aracatuba, Bauru, Marilia, Presidente Prudente, and Sao Jose do Rio Preto, state of Sao Paulo,1999 to 2013.

Figure 4 (A) represents the distribution of HVL-related deaths per area and Figure 4 (B), the mortality rate and the existing highway network in the area, whose rate was equal to or greater than the median (0.17 deaths per 100 thousand inhabitant-years). Despite having lower values than the ones seen in Figure 3 (A and B), the behavior of the number of deaths and the mortality rate was, respectively, similar to the behavior of number of HVL cases and the incidence rate.

**Figure 5:**
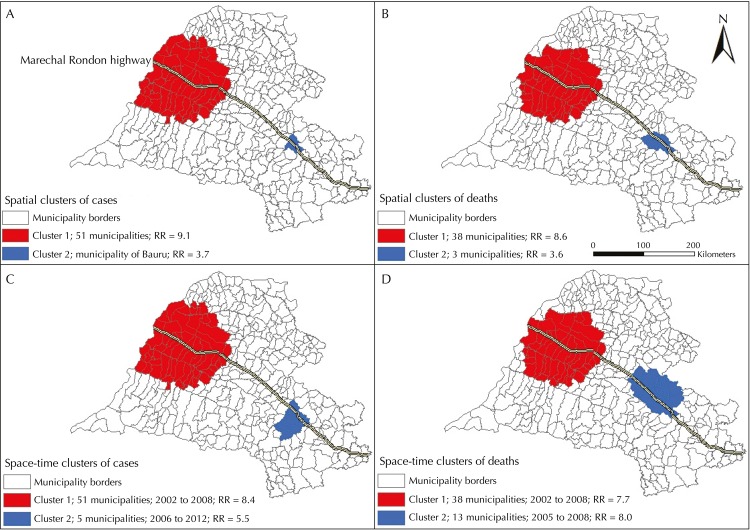
Spatial clusters of cases (A) and deaths (B) and space-time clusters of cases (C) and deaths (D) from human visceral leishmaniasis. Health care regions of Aracatuba, Bauru, Marilia, Presidente Prudente, and Sao Jose do Rio Preto, state of Sao Paulo, 1999 to 2013.

Using spatial and space-time scan statistic tools allowed detecting two significant spatial clusters of HVL cases (Figure 5, A). The first comprised 51 municipalities from the health care regions of Araçatuba (Araçatuba included), Marilia, Presidente Prudente, and Sao Jose do Rio Preto; out of these, 47.0% belonged to the first region, the one with the oldest reports. The second significant cluster only included the municipality of Bauru, the first one to detect human autochthonous cases of visceral leishmaniasis in the region of Bauru (Figure 2, B).

Regarding deaths, two spatial clusters were identified. The first one comprised 38 municipalities from the health care regions of Aracatuba (Aracatuba included), Marilia and Presidente Prudente, with a predominance of 13 (34.0%) in the first region. The second significant spatial cluster comprised Bauru and two other neighbor municipalities. The RRs of catching HVL and dying from it in the spatial clusters are shown in Figure 5 (A and B).

The space-time analysis of HVL cases found two clusters (Figure 5, C). The first one comprised 51 municipalities from all health care regions. With the exception of Bauru, it was spatially identical to the spatial cluster that was found in this area, and it took place between 2002 and 2008. The second space-time cluster of cases involved five municipalities in the health care region of Bauru (Bauru included), and it took place between 2006 and 2012.

The first significant space-time cluster of deaths was identical, from a spatial perspective, to the spatial cluster of deaths, and it took place between 2002 and 2008; the second one comprised 13 municipalities in the region of Bauru (Bauru included) and took place between 2005 and 2008. The RRs of catching HVL and dying from HVL in the space-time clusters are shown in Figure 5 (C and D).

## DISCUSSION

HVL incidence in the studied area has been similar to the two cases per 100 thousand inhabitant-years found in Brazil since the stabilization of rates that took place as of 2001^a^. Regarding lethality, the value that was obtained for the studied area falls within the ones recorded in Sao Luis (Maranhao), Campo Grande (Mato Grosso do Sul), and Belo Horizonte (Minas Gerais)^1^ ^,^ ^4^ ^,^ ^15^.

The highest incidence rates found for male individuals and children of between zero and four years of age corroborate the findings of other studies, in which the higher incidence rates found for males have been attributed to occupational issues and the ones for children have been ascribed to their incomplete immune system development^2^ ^,^ ^5^ ^,^ ^25^ ^,^ ^26^.

Visceral leishmaniasis was considered an endemic that was typical to rural areas until the 1970s, with almost all cases being found the Brazil’s Northeast region. From the mid 1980s onwards, it has also begun to take place in urban areas and to spread to other regions^13^ ^,^ ^20^ ^,^ ^a^. They are studied here is an example of this process, with almost all reported autochthonous cases from 1999 on taking place in urban regions.

The changes in the epidemiological characteristics of this zoonosis, especially its occurrence in urban areas and its expansion, are related to migration and environmental changes, besides other anthropic factors^1^ ^,^ ^22^, and to the complex management of vectors and dogs^19^; control strategies consequently have not been sufficient to avoid it^11^.

Migration, which is generally triggered by socioeconomic matters, has been pointed out as one of the factors responsible for the dissemination of the vector and the spread of visceral leishmaniasis throughout Brazil. In Sao Luis, such process was triggered by the implementation of an industrial district^16^. Similar examples took place in Varzea Grande (Mato Grosso do Sul)^18^, Brasilia^8^, and Aracaju (Sergipe)^12^. These migration processes, which, in the studied area, are especially originated in Minas Gerais and related to the replacement of cattle herds by sugarcane plantations^3^, generate new anthropic changes, which have been creating favorable conditions for the evolution of visceral leishmaniasis. Poor sanitation infrastructure and housing conditions; exposure to infected dogs^17^ ^,^ ^24^; adaptive factors of the vector; construction sites, and heavy flow of people, products, and services in highways and railways have also been related to the expansion and urbanization of visceral leishmaniasis in Brazil^1^ ^,^ ^3^ ^,^ ^17^ ^,^ ^22^ ^,^ ^27^.

The construction of Sao Paulo’s portion of Bolivia-Brazil gas pipeline and the reactivation of Novoeste railway in Sao Paulo brought many workers from other states and countries to the region, who were constantly on the move as a result from going to different construction sites and transporting machines and equipment^1^. This migration of people also possibly triggered the migration of dogs infected by leishmania^17^ ^,^ ^24^. In Mato Grosso do Sul, the construction of its gas pipeline was pointed out as responsible for disseminating the vector and HVL^1^ ^,^ ^6^.

The flow of people, goods, and services in railways, and especially in highways, seems to have a highlighted role in the spread of visceral leishmaniasis in Sao Paulo. Aracatuba and Birigui, located in the health care region of Aracatuba – the first municipalities to report HVL cases, are on the way of Marechal Rondon highway, the main connecting route between the regions of Aracatuba and Bauru and between them and the state capital. As observed by Cardim et al.^7^, the course of dispersion and spread of HVL, from the region of Aracatuba, has followed the route or Marechal Rondon highway eastwards. From this highway, it followed the course of the transverse highways and other radial highways, reaching municipalities in the regions of Bauru, Marilia, Presidente Prudente, and Sao Jose do Rio Preto.

In the health care regions of Aracatuba and Bauru, the first HVL cases were found in the cities who lend their names to the regions and in the region of Sao Jose do Rio Preto, in Jales, in the microregion of the same name. These places, along with their highways, are highlighted as places with a heavy flow and, therefore, with higher chances of having visceral leishmaniasis and its vector. This was not observed in the regions of Presidente Prudente and Marilia, but the influence of the highway network could also be noticed. Dracena and Ouro Verde, in the first region, are close to the Comendador João Ribeiro de Barros Highway and Guaranta; the second region is intercepted by Marechal Rondon highway.

The role played by the main cities in the regions in the spread of visceral leishmaniasis was highlighted in the Kernel maps of cases and deaths. The ellipses centered around Aracatuba indicate that this city is supposedly the main focus in the spread of the disease in Sao Paulo, once it was the first city to identify the presence of the vector^10^ and to report canine and human cases. A second ellipse was formed in the health care region of Bauru with epicenter in Bauru. It may be inferred to have been the main focus of the spread of the disease in this region. Identifying Aracatuba and Bauru as the epicenters of the spread of HVL is in agreement with the fact they are regional centers that trigger, mainly by their highways, a heavy flow of people, goods, and services. This same role as a regional center influencing the spread process of HVL was identified in Varzea Grande^18^.

The observation of Kernel ratio maps for incidence and mortality due to HVL showed that the ellipses formed kept indicating Bauru as a spreading spot of the disease. However, the ellipses in the region of Aracatuba were changed, and their epicenter was observed in Mato Grosso do Sul’s border, highlighting the role of this state in the disease epidemiology in Sao Paulo^1^.

In the maps obtained by Kernel and Kernel ratio, Marechal Rondon highway may be characterized as the main axis of the ellipses found. We also found it to be the core axis in all spatial and space-time clusters identified. As pointed out by Cardim et al.^7^, these results corroborate the role played by this highway in the spreading process of the disease in Sao Paulo. Antonialli et al.^1^ and Mestre and Fontes^17^, when respectively studying visceral leishmaniasis in Mato Grosso do Sul and in Mato Grosso, also highlighted the role played by the highways, among other factors, in the spread of the disease. Besides Marechal Rondon highway, Novoeste railway, which runs parallel to the former, may have also favored the spread of HVL in Sao Paulo.

The fact that the Novoeste railway and Marechal Rondon highway are connected to Mato Grosso do Sul by the municipality of Castilho, in the state of Sao Paulo, and Tres Lagoas, in Mato Grosso do Sul, reinforce the influence of railway and highway networks in the spreading process of HVL in Sao Paulo. At that point, Marechal Rondon connects to BR-262, following its course as far as Corumba (Mato Grosso do Sul), a municipality that has been endemic to HVL since 1982. Corumba is located in the border between Brazil and Bolivia, and this easy connection between the two states and the foreign country has already been mentioned^1^ as one of the factors responsible for the spread of HVL in Mato Grosso do Sul, because it favors, among other aspect, the migration process.

The space-time evaluation, by identifying areas of higher risk simultaneously in space and time, showed that, especially in the region of Aracatuba, HVL has spread more between 2002 (three years after the identification of human autochthonous cases) and 2008, lasting for six years. In the region of Bauru, in turn, the most intense spread took place between 2006 (also three years after the first cases were identified in this health care region) and 2012, also lasting for six years. The coincidental duration of the two space-time clusters, with non-simultaneous occurrence in time, is in agreement with the cyclic aspect expected for this kind of disease, leading us to ask whether the tool used may be useful to quantify this process, a hypothesis which should be tested in future studies.

Among the limitations in this study are the use of secondary data sources and the fact that these data result from the reports available, which may not correspond to the total number of cases. However, by the use of GIS and spatial analysis tools, it was possible to describe the spread of visceral leishmaniasis in Sao Paulo, to identify risk areas, and to point out possible determining factors of this process, especially the role played by highways. These results, if considered, may enable the improvement of initiatives for monitoring and controlling visceral leishmaniasis^9^ ^,^ ^21^.

## Footnotes

a World Health Organization. Control of leishmaniases. Geneva: WHO; 2010. (Technical Report Series, 949).

b Ministério da Saúde, Secretaria de Vigilância em Saúde, Departamento de Vigilância Epidemiológica. Manual de vigilância e controle da leishmaniose visceral. Brasília (DF); 2006.

c Secretaria de Saúde do Estado de São Paulo, Superintendência de Controle de Endemias, Coordenadoria de Controle de Doenças. Manual de vigilância e controle da leishmaniose visceral americana do Estado de São Paulo. São Paulo (SP); 2006.
